# The m^6^A epitranscriptome on neural development and degeneration

**DOI:** 10.1186/s12929-021-00734-6

**Published:** 2021-05-27

**Authors:** Ya-Ping Yen, Jun-An Chen

**Affiliations:** Institute of Molecular Biology, Academia Sinica, Taipei, 11529 Taiwan

**Keywords:** m^6^A, Epitranscriptome, RNA, Neural development, Neurodegeneration

## Abstract

*N*^6^-methyladenosine (m^6^A) is the most prevalent, conserved, and abundant RNA modification of the mRNAs of most eukaryotes, including mammals. Similar to epigenetic DNA modifications, m^6^A has been proposed to function as a critical regulator for gene expression. This modification is installed by m^6^A methylation “writers” (Mettl3/Mettl14 methyltransferase complex), and it can be reversed by demethylase “erasers” (Fto and Alkbh5). Furthermore, m^6^A can be recognized by “readers” (Ythdf and Ythdc families), which may be interpreted to affect mRNA splicing, stability, translation or localization. Levels of m^6^A methylation appear to be highest in the brain, where it plays important functions during embryonic stem cell differentiation, brain development, and neurodevelopmental disorders. Depletion of the m^6^A methylation writer *Mettl14* from mouse embryonic nervous systems prolongs cell cycle progression of radial glia and extends cortical neurogenesis into postnatal stages. Recent studies further imply that dysregulated m^6^A methylation may be significantly correlated with neurodegenerative diseases. In this review, we give an overview of m^6^A modifications during neural development and associated disorders, and provide perspectives for studying m^6^A methylation.

## Introduction

**Fig. 1:**
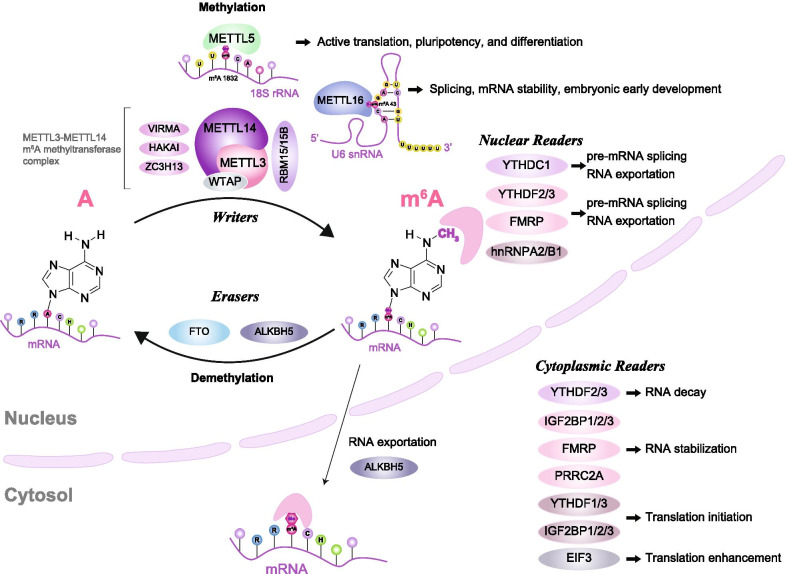
m^6^A-associated proteins and molecular pathways. *N*^6^-methyladenosine (m^6^A) modifications have important roles in a series of RNA-centric regulatory mechanisms. The illustration depicts the roles of m^6^A “writer”, “eraser” and “reader” complexes in regulating mRNAs. m^6^A biogenesis in mammalian cells (nucleus) is catalysed by a core methyltransferase complex comprising METTL3, METTL14 and WTAP, and it can be reversed by m^6^A demethylases (FTO or ALKBH5). METTL5 and METTL16 have recently been reported as additional writers to catalyze the addition of m^6^A on the 3′UTR of 18S rRNA and U6 snRNA, respectively. m^6^A can be recognized by reader proteins in both the nucleus and cytoplasm. For example, YTHDF2/3 can participate in post-transcriptional regulation by recruiting different complexes to m^6^A sites. m^6^A-modification of mRNAs can impact RNA nuclear splicing, export, stability, trafficking, and translation efficiency. METTL, methyltransferase-like; WTAP, WT1-associated protein; RBM15, RNA-binding motif protein 15 or its paralog RBM15B; VIRMA, vir-like m^6^A methyltransferase-associated; HAKAI, E3 ubiquitin-protein ligase Hakai (also known as CBLL1, cbl proto-oncogene-like 1); ZC3H13, zinc finger CCCH-type containing 13 proteins; FTO, fat-mass and obesity-associated protein; ALKBH5, alkB homolog 5; YTHDF, YT521-B homology domain family; FMRP, fragile X mental retardation protein; hnRNPA2/B1, heterogeneous nuclear ribonucleoprotein A2/B1; PRRC2A, proline rich coiled-coil 2A; IGF2BP1/2/3, insulin like growth factor 2 mRNA binding protein 1/2/3; elF3, E74-like factor 3

Gene expression and cell division are controlled by genetic and epigenetic regulation. Abnormal genetic changes (such as gene mutation, deletion, or amplification, as well as chromosomal translocation or epigenetic abnormalities such as DNA methylation or histone modification) may result in developmental defects or diseases. In recent years, RNA modifications have gained increasing attention for their largely unexplored roles in gene regulation (i.e., RNA epitranscriptomics) [1]. Since the 1950s, over 100 types of RNA modification have been identified. With the power of new high-throughput sequencing methods, a diversity of mRNA modifications—including *N*^6^-methyladenosine (m^6^A), *N*^1^-methyladenosine (m^1^A), 5-methylcytosine (m^5^C), 5-hydroxymethylcytosine (hm^5^C), and pseudouridine (ψ)—have been revealed in various organisms [2]. Among them, dynamic and reversible m^6^A mRNA modification, discovered in the 1970s [3], arguably represents the most widely distributed form of mRNA modification in mammals. m^6^A is deposited by the m^6^A methyltransferase complex (termed a “writer”) that comprises Mettl3 (methyltransferase-like 3), Mettl14 (methyltransferase-like 14), Wtap (Wilms tumor 1-associated protein), Virma (VIR-like m^6^A methyltransferase associated), RBM15 (RNA-binding motif protein 15) and its paralogue (RBM15B) [4, 5]. Conversely, m^6^A can be removed by m^6^A demethylases (termed “erasers”), such as Fto (fat mass and obesity-associated protein) and Alkbh5 (alkB homolog 5). m^6^A tagging has multiple functions, including in mRNA splicing, stability, nuclear export, localization, translational efficiency activation, and decay of target mRNA stability (Fig. 1) [6–8]. Notably, m^6^A deposition manifests at the highest levels in the central nervous system (CNS), where it plays major roles in embryonic stem cell differentiation, brain development, and neurodevelopmental disorders [9, 10]. Recent studies have shown that constitutive knockout of *Mettl14*—a key element of the m^6^A methyltransferase complex—is embryonically lethal, whereas conditional knockout (cKO) of *Mettl14* in neural progenitor cells disrupts cortical development and leads to premature death in mice [11, 12]. Interestingly, levels of m^6^A are relatively low in mouse brain tissue during embryogenesis, but drastically increase by adulthood [10], suggesting that m^6^A modification plays a unique role in the adult brain. This scenario also raises the possibility that m^6^A might play an important role in adult RNA homeostasis and that associated imbalances might lead to onset or progression of neurodegeneration. This notion is supported by studies showing that the m^6^A demethylase Fto plays an important role in learning and behavior [13–15]. In addition to m^6^A’s functions in the CNS, Mettl3*,* another component of the m^6^A methyltransferase complex, has been shown to affect plant growth, yeast meiosis, mammalian metabolism, synaptic signaling, stem cell self-renewal, and differentiation [16]. Furthermore, deletion of *Mettl3* from cardiomyocytes reduces m^6^A levels, resulting in long-term loss of normal cardiac homeostasis and function in adult mouse heart [17]. Thus, overall, m^6^A modification plays versatile roles during embryonic development, as well as functioning in adult homeostasis, but its most prominent roles lie in the CNS.

**Fig. 2:**
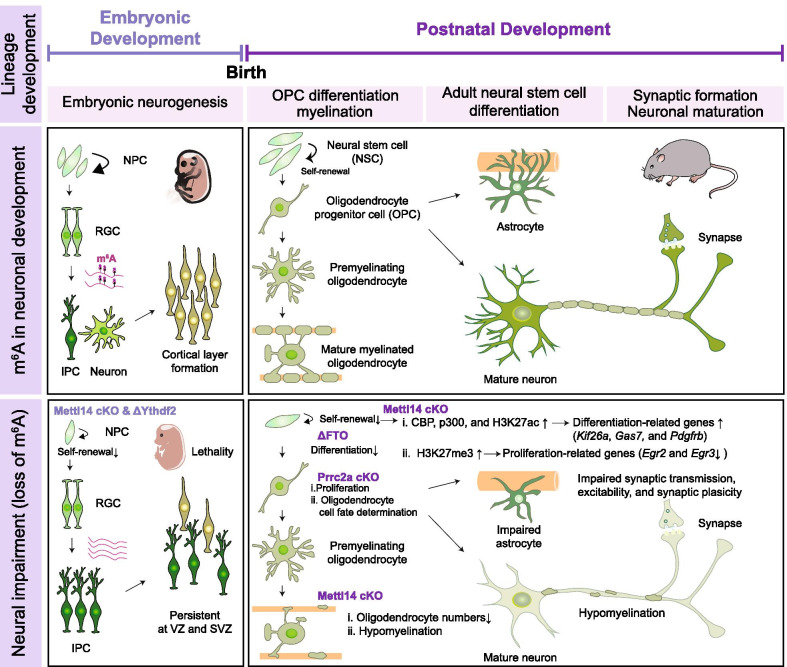
The roles of m^6^A in the developing central nervous system. m^6^A RNA modifications play important roles in regulating neural development, including embryonic neurogenesis, OPC differentiation, myelination, adult NSC differentiation, synaptic formation and neuronal maturation. The panels summarize the physiological functions of m^6^A during neural development, as revealed by loss-of-function studies on m^6^A-associated proteins. NPC, neural progenitor cell; RGC, radial glial cell; IPC, intermediate progenitor cell; NSC, neural stem cell; OPC, oligodendrocyte progenitor cell; CBP, CREB binding protein; H3K27ac, histone H3 lysine 27 acetylation; H3K27me3,  histone H3 lysine 27 trimethylation

As many previous reviews have illustrated the importance of m^6^A modification of mRNAs during early embryonic development [12, 18, 19], here we provide an overview of current progress on m^6^A epitranscriptomic regulation of neural development and its biological implications. In addition, we review the roles of m^6^A during adult neurogenesis and potential consequences for neurological disorders (Fig. 2 and Table 1). Finally, we present our perspectives for future research directions of m^6^A in neural development and degeneration.

**Table 1:** Roles of m^6^A methylation in the developing CNS

Roles of m^6^A methylation in developing CNS						
Lineage development	m^6^A methyltransferase component involved	Type	m^6^A-methylated target mRNAs	Mouse models	Neural development function affected	Refs.
Cortical neurogenesis	Mettl14	Writer	Neuronal differentiation-related transcription factors (*Pax6*, *Sox1*, *Sox2*, *Emx2*, *Neurog2*, and *Neurogenin 2*), and proneural genes (*Neurod1/2*)	*Nestin-cre;Mettl14*^*f/f*^	1. Temporal specification and cell-cycle progression of NPCs 2. Transcriptional regulation in NSCs 3. Cell-cycle progression of cortical neural progenitors	[12]
			Histone acetyltransferase (CBP/p300)	*Nestin-cre;Mettl14*^*f/f*^	1. Self-renewal of NSCs	[19]
	Ythdf2	Reader	Positive regulation of cell differentiation and GTPase activity Negative regulation of JAK-STAT cascade genes (*Flrt2/3*, *Ptprd*, and *Lrrtm1/4*)	*Ythdf2* cKO	1. Clearance of negative regulators of neurogenesis	[18]
Neural differentiation	Fmrp	Reader	Nuclear retention of m^6^A-tagged Fmrp target mRNAs	*Fmrp* KO	1. Promotion of mRNA nuclear export during neural differentiation	[74]
Oligodendrocyte development and myelination	Mettl14	Writer	Oligodendrocyte lineage genes (*Ptprz1*, *Gsn*, and *Map2*)	*Olig2-cre;Mettl14*^*f/f*^ and *CNP-Cre;Mettl14*^*f/f*^	1. Differentiation and myelination of OPCs	[57]
	Prrc2a	Reader	Oligodendroglial transcriptional factor (*Olig2*)	*Nestin-Cre;Prrc2a*^*f/f*^	1. Proliferation and differentiation of OPCs 2. Stabilization of *Olig2* mRNA for oligodendrocyte maturation	[56]
Cerebellar development	Alkbh5	Eraser	Loss of methylated RNAs has been linked to altered cell division (*Cenp*e), cell cycle (*Cdca2*), and cell projection organization (*Erbb4*) genes Hypermethylated RNAs are related to metabolic processes (*Ccr5*), ion transport (*Camk2g*), and and axon guidance (*Rora*)	*Alkbh5* KO	1. Proliferation and differentiation in the cerebellum 2. Disrupted RNA metabolism of a subset of cell fate determination genes 3. Defected cerebellar development under hypobaric hypoxia	[60]
Neurogenesis in the brain	Fto	Eraser	Loss of *Fto* led to altered expression of several brain-related neurotrophic factors	*Fto* KO	1. Decreased brain size and body weight 2. Reduced pool of NSCs in the SGZ region 3. Reduced proliferation and neuronal differentiation in NSCs 4. Impairment of learning and memory	[52]
Hippocampal neurogenesis	Ythdf1	Reader	Synaptic plasticity transcripts (*Gria1*, *Grin1*, and *Camk2a*)	*Ythdf1* KO	1. Learning and memory 2. Basal transmission and long-term potentiation at synapses in the hippocampus	[61]
Axon guidance	Ythdf1	Reader	Axon guidance receptor *Robo3.1*	*Ythdf1* KO	1. Axon guidance in spinal commissural axons	[75]
Axon regeneration	Ythdf1	Reader	Axon regeneration-related genes (*Tet3* and *Gadd45a*)	*Ythdf1* KO	1. Axon regeneration in the peripheral nervous system 2. Translation of injury-induced protein 3. Axon regeneration in adult mouse dorsal root ganglions	[11]

## *N*^6^-methyladenosine (m^6^A)

m^6^A is known to be chemically stable, but the methyl modification is dynamic due to low cellular abundance of mRNAs (representing 2–5% of total RNA), with 0.2–0.5% of total mRNA adenines being m^6^A-modified [7, 9, 31, 32]. Although few genes are m^6^A-modified, this modification appears to play a significant regulatory role in gene expression [33]. Over the past decade, multiple studies have shown that Mettl3/Mettl14 together with other components of the m^6^A methyltransferase complex can dynamically exert m^6^A mRNA modifications on mRNAs or long non-coding RNAs to regulate mRNA stability, translation efficiency, localization, and splicing (Fig. 1). An early study provided the first evidence that reversible post-transcriptional RNA modifications enact regulatory functions to fine-tune the structure and function of RNAs [1]. In that study and a subsequent one, a m^6^A demethylase (Fto) was shown to catalyze oxidative m^6^A demethylation of nuclear RNA [1, 34]. Later, another m^6^A demethylase, Alkbh5, was shown to affect mouse fertility and spermatogenesis [35]. Following these breakthrough findings, dynamic m^6^A methylation and gene regulation have gained increasing attention. Several insightful reviews have illustrated details of the molecular pathways underlying m^6^A modification [36–41]. In this review, we focus on providing a brief overview of recent exciting discoveries on how the m^6^A epigenome regulates neural development and degeneration.

To understand the fundamental functions of m^6^A in mRNAs, it is necessary to determine the positions of m^6^A sites in gene transcripts. In 2012, two groups independently reported on antibody-based m^6^A RNA immunoprecipitation sequencing (RIP-Seq/MeRIP-Seq) techniques and subsequent mapping of the m^6^A transcriptome [10, 31]. Comprehensive analyses of those sequencing data revealed that primary m^6^A sites are enriched in the regions of translational stop codons and in 3′ untranslated regions (3′UTR), suggesting m^6^A may serve a role in mRNA translation [10, 31, 42]. Moreover, the consensus motif RRACH (in which R represents A or G, and H represents A, C or U) mediates most m^6^A depositions. Although m^6^A does not alter Watson–Crick base pairing, the m^6^A modification promotes destabilization of A/U pairings and alters RNA secondary structure [43]. As the frequency of this consensus motif is much higher in the genome than m^6^A occurrence, additional sequences or RNA structures may also play as yet unidentified roles in determining methylation sites. For instance, recent reports have demonstrated that m^6^A reshapes protein and RNA binding, thereby affecting mRNA secondary structure [44, 45].

By revealing m^6^A deposition on mRNAs via MeRIP-Seq, m^6^A abundance has been demonstrated as both tissue-specific and species-specific [46]. Brain tissue displays the highest levels of m^6^A deposition, with > 30% of its transcripts being m^6^A-modified [24]. Detailed characterizations have further highlighted that m^6^A occurs within various types of RNA other than mRNA, such as tRNA, rRNA, non-coding RNA (ncRNA), and snRNAs [5, 22, 27, 30, 45, 47–49], but the consensus motifs in those RNAs differ from those in mRNA. For example, m^6^A uses the *U6* snRNA as substrate to form 3′-stem loop structures in vitro [30, 50]*.* Therefore, m^6^A may serve some unique functions in mRNAs that are different from those in other RNA types, perhaps being cell-type-dependent and tissue-specific. Collectively, current evidence indicates a functional significance for m^6^A modifications of RNA. However, the exact molecular mechanisms underlying m^6^A methylation sites in consensus motifs of mRNAs remain to be elucidated.

## m^6^A epitranscriptomics in neural development

m^6^A has been reported to be the most abundant epitranscriptomic mark for mRNA modification in eukaryotes. Emerging studies of the CNS have shown that most mRNAs present low levels of m^6^A modification in the brain from the embryonic to postnatal stages, but levels dramatically increase in adulthood [10, 24]. The importance of m^6^A in the CNS seems to be conserved from flies to mammals. In Drosophila, the m^6^A epitranscriptome is dynamic and remarkably enriched in early embryogenesis, but it declines dramatically two hours after fertilization. Then, m^6^A remains at a low level throughout the rest of embryogenesis and during early larval stages. During the third larval instar, m^6^A levels increase again, reaching a peak in pupal phases. Although overall levels of m^6^A decrease in adult Drosophila, it remains substantially elevated in head and ovary tissues. When the *Mettl3* orthologue, *Ime4*, is knocked out, the mutant flies manifest reduced lifespan, severe behavioral defects, and altered neural gene expression. Recent works have also shown that m^6^A regulates neural development and brain function in mouse models [12, 39]. In human, two studies have reported that children with homozygous missense mutations in *FTO* (m^6^A demethylase) presented severe neurodevelopmental disorders, including microcephaly, developmental delay, behavioral abnormalities, dysmorphic facial features, hypotonia, and various other phenotypic abnormalities, suggesting an essential, yet unexplored, role for m^6^A RNA modification in brain development [21, 25]. Thus, neural development represents one of the best paradigms for elucidating the functional significance of m^6^A modification.

## Role of m^6^A in regulating neurogenesis

Embryonic neurogenesis is coordinated between neural progenitor cell (NPC) proliferation and cell fate specification. NPCs differentiate into various neural and glial cell subtypes before migrating to their final destinations in the CNS [20, 29]. In addition, long non-coding RNA represses progenitor genes and maintains neural cell fate identity representing a highly organized topographic migratory process from embryonic to postnatal stages [51]. Notably, Yoon et al. showed that *Mettl3* is highly enriched during the early stage of neurogenesis [12], whereas *Fto* is expressed more prominently during the later stage of neurogenesis [52]. Conditional *Mettl14* knockout (cKO) in mouse NPCs using *Nestin*-Cre impairs NPC differentiation, prolongs cell cycle progression of radial glia, and extends cortical neurogenesis into postnatal stages [12]. Similar phenomena have also been observed upon knockdown of another m^6^A writer, *Mettl3* [12]. Prolongation of cell cycle progression in neural stem cells (NSCs) upon loss of either *Mettl3* or *Mettl14* delays production of upper-layer neurons in postnatal mouse cortex. Human induced pluripotent stem cell (iPSC)-derived brain organoids were also used to confirm that m^6^A regulates NPC cell cycle progression in a human context [12]. Further comparison of the m^6^A-seq data from human forebrain organoids and E13.5 mouse forebrains revealed that m^6^A-modified transcripts are conserved and have distinct m^6^A epitranscriptomic landscape features. Interestingly, many transcripts encoding transcription factors are m^6^A-tagged, such as *Pax6*, *Sox1*, *Sox2*, *Emx2*, and *Neurog2/Neurogenin 2* [12]*.* Gene ontology (GO) analysis of the m^6^A-modified transcripts in both mouse and human revealed enrichment of genes related to neurogenesis, neuronal differentiation, and development. Furthermore, disease ontology analysis of those transcripts uniquely m^6^A-tagged in human highlighted enrichment for neurodevelopmental diseases such as schizophrenia and autism, implying that m^6^A may contribute to human psychiatric or neural disorders. Thus, Yoon et al. have provided the first proof-of-principle of a m^6^A epitranscriptomic mechanism contributing to conserved transcriptional coordination during mammalian cortical neurogenesis.

Similarly, Wang et al. used a mouse genetic model to conditionally inactivate *Mettl14* in embryonic NPCs, which also revealed that *Mettl14* is required for NPC proliferation, with consequent loss of m^6^A in the CNS slowing NPC cell cycle progression so that the NPCs remained in an undifferentiated state [19]. To further examine the consequences of m^6^A loss, they systematically characterized *Mettl14* cKO NPCs in vitro and observed that loss of m^6^A reduced NSC proliferation and resulted in precocious NPC differentiation in vitro. Furthermore, cortical radial glial cells (RGCs) in the brain were found to be smaller and numbers of late-born neurons were reduced in the *Mettl14*-cKO mutant mice. Profiling of histone modifications upon m^6^A loss via *Mettl14* knockout revealed increased histone H3 acetylation at lysine 27 (H3K27ac), histone H3 trimethylation at lysine 4 (H3K4me3), and histone H3 trimethylation at lysine 27 (H3K27me3) in cell-proliferation related genes. Most of the changes in histone levels could be rescued by treating cells with H3K27ac or H3K27me3 inhibitors, indicating that m^6^A RNA methylation serves an essential function in regulating NSC self-renewal via m^6^A*-*mediated histone modification during NSC-related gene expression. These changes in histone modification were also partially attributable to m^6^A*-*mediated destabilization of transcripts of the histone acetyltransferase CBP (CREB binding protein) and p300, both of which were stabilized upon loss of m^6^A. This study by Wang et al. has provided new insights into crosstalk between RNAs and histone modification. Importantly, their study also indicates that different m^6^A-regulated histone marks coordinate active/repressive gene expression, implying that m^6^A-regulated active and repressive histone modifications work synergistically to ensure an NSC differentiation program.

Both the Yoon et al. and Wang et al. studies utilized conditional knockout of the m^6^A writer *Mettl14* in the developing forebrain as an experimental paradigm. Although the mechanisms revealed by these two studies as underlying m^6^A function differ, both studies revealed similar phenotypes, such as delayed NPC cell cycle progression. Another study has provided evidence that the m^6^A “reader” protein Ythdf2 also participates in cortical neurogenesis [18]. *Ythdf2* knockout mice die at late embryonic developmental stages. Furthermore, *Ythdf2*-deficient NSCs display diminished proliferation and differentiation, and neurons derived from *Ythdf2*-deficient NSCs have shorter neurites and are vulnerable to oxidative stress. When Li et al. examined the proliferative and differentiation capabilities of neural stem/progenitor cells (NSPCs), they observed a dramatically reduced overall cortical thickness of *Ythdf2*-deficient embryonic forebrains [18]. Furthermore, NPSC self-renewal and spatiotemporal generation of neurons and other cell types were severely negatively impacted in embryonic neocortex upon loss of *Ythdf2*. Since neurite outgrowth is critical for neuronal development and maturation, as well as synapse formation, the abnormal neurite branching and extension presented by *Ythdf2*-deficient neurons might contribute to defective neurogenesis during neural development. Consistent with the findings of Yoon et al. [12], deletion of *Mettl14* and *Ythdf2* led to enlarged ventricles and decreased cortical thickness, respectively [18]. As Ythdf2 is known to bind m^6^A-methylated mRNAs and promotes mRNA decay [8, 53], Li et al. further demonstrated increased expression of m^6^A-tagged gene transcripts associated with neural development and cortical neuron differentiation upon loss of *Ythdf2*-mediated RNA degradation. This scenario adds an additional layer of m^6^A-dependent control of the neural development-related mRNA targets recognized by Ythdf2 and that modulate neural development. Thus, fundamentally, the Ythdf2-mediated functions of m^6^A epitranscriptomic regulation are not only essential for post-transcriptional regulation of the maternal transcriptome and oocyte competence [54], but are also crucial for regulating cortical neurogenesis during embryonic neural development. However, how the m^6^A reader Ythdf2 interprets m^6^A epitranscriptomic regulation of cortical neurogenesis for neural development and differentiation is not entirely deciphered. Since a battery of m^6^A “reader” proteins have already been identified in various cell types [23, 28, 46, 55], further neuron-specific activities could multiply the highly variable functions of m^6^A during neural development.

## Role of m^6^A in oligodendrocytes

Differential m^6^A methylation may also play important roles in oligodendrocyte development and CNS myelination [56, 57]. Xu et al. characterized the pathological consequences of *Mettl14* ablation for oligodendrocyte lineage progression [56, 57]. In that study, numbers of mature oligodendrocytes were reduced in the corpus callosum of both *Olig2-Cre;Mettl14*^*f/f*^ and *CNP-Cre;Mettl14*^*f/f*^ mutant mice. Although *Olig2-Cre;Mettl14*^*f/f*^ mutant mice exhibited a relatively normal postnatal phenotype, they began displaying occasional hindlimb flexion, slight ataxia, and mild tremors after 6 months. In addition, *CNP-Cre;Mettl14*^*f/f*^ mutant mice started to display tremors and hindlimb clenching at ~ 4 months of age, with a gradual worsening of the ataxic phenotype after onset. Both the corpus callosum and optic nerve were hypomyelinated in *Olig2-Cre;Mettl14*^*f/f*^ and *CNP-Cre;Mettl14*^*f/f*^ mutant mice, as revealed by electron microscopy at postnatal day 18 (P18). This observation indicates that m^6^A methylation is important for oligodendrocyte development and CNS myelination. The severe developmental phenotypes observed in vitro and in vivo are consistent with oligodendrocyte lineage progression being controlled by dynamic changes in m^6^A modification of numerous transcripts. Mechanistically, *Mettl14* deletion was shown to differentially alter *Nfasc155* alternative splicing and expression in *Olig2-Cre;Mettl14*^*f/f*^ mutant mice. Thus, m^6^A might act in generating functional isoforms of myelin proteins via alternative splicing to ensure precise oligodendrocyte lineage progression.

Consistent with the study by Xu et al. [56, 57], Wu et al. reported a novel m^6^A “reader” protein, proline rich coiled-coil 2 A (Prrc2a), which modulates oligodendrocyte progenitor cell (OPC) specification, proliferation, fate determination and CNS myelination, affirming the importance of m^6^A modification in the glial lineage [56]. Genetic deletion of *Prrc2a* from brain of *Nestin-Cre;Prrc2a*^*f/f*^ and *Olig2-Cre;Prrc2a*^*f/f*^ mutant mice led to hypomyelination in the corpus callosum. Moreover, the mutant mice displayed locomotive and cognitive disabilities, as well as decreased lifespan, though neurogenesis was not affected. When Wu et al. compared Prrc2a binding targets that contain m^6^A peaks with the downregulated differentially-expressed genes (DEG) of *Olig2-Cre;Prrc2a*^*f/f*^ mutant mice, they found that Prrc2a binds and stabilizes m^6^A-methylated transcripts of oligodendrocyte transcription factor 2 (*Olig2*), a key oligodendroglial lineage-determining transcription factor. *Prrc2a*-knockdown reduced both mRNA and protein levels of Olig2, whereas *Prrc2a* overexpression enhanced them, indicating that *Prrc2a* post-transcriptionally regulates *Olig2* expression. Additionally, knockout of the m^6^A demethylase *Fto* recapitulated the phenotype of enhanced *Olig2* expression displayed upon *Prrc2a* overexpression. That study has revealed yet another function for m^6^A RNA modification associated with the myelination process of oligodendrocytes. Thus, apart from its essential roles in neurons, m^6^A also participates in myelination by regulating oligodendrocyte development.

## Role of m^6^A in adult brain development

In addition to the roles of *Mettl14* and m^6^A in embryonic neural development, m^6^A also functions in adult mouse brain. One prominent activity is its regulation of synaptic function and stress-induced responses. In this scenario, most cortical genes expressed in adult mouse are m^6^A-methylated, with m^6^A also being enriched in synaptic transcripts [24]. Moreover, *Mettl14*, *Fto*, and *Ythdf1/2/3* are enriched during dendritic development of cortical neurons [58]. In hippocampal neurons, more than 1000 transcripts are m^6^A-methylated, which related to synaptic organization, assembly, maturation, and transmission modulation [58]. *Mettl14* deletion reduced m^6^A methylation levels in synaptic plasticity-related transcripts that are correlated with impaired neuronal excitability levels, without altering cell numbers or morphology [59]. It also increased neuronal excitability, reduced spike frequency adaptation, and profoundly impaired striatal-mediated behaviors, suggesting that m^6^A is important for maintaining normal striatal function in adult mice [60]. The functions of m^6^A in the adult CNS have been further revealed by two additional sets of studies. First, increased m^6^A methylation of synaptic transmission-related mRNAs has been observed in the midbrain of *Fto*-deficient mice [13]. Second, m^6^A mRNA methylation was shown to facilitate learning and memory formation in mouse hippocampus at postnatal days 30 and 120. Upon loss of *Ythdf1*, learning and memory defects, as well as functional deficits in hippocampal excitatory synaptic transmission, were manifested by promoting the translation of m^6^A-modified transcripts [58, 61]. These observations highlight critical functions for m^6^A in neural circuit formation, especially for synaptic plasticity, and highlight a new aspect of m^6^A mRNA methylation-dependent translational regulation. Overall then, m^6^A is an important epitranscriptomic RNA modification that is highly expressed during neurogenesis in the brain, from embryonic to adult stages. m^6^A methylation broadly affects the CNS, acting in NSC self-renewal, glioma cell proliferation, brain development, synaptic growth, learning and memory. Thus, intuitively, it is not surprising that m^6^A might play a prominent role in neurological disorders and diseases.

## Neurological disorders

Although several studies have shown that the m^6^A epitranscriptome is important for neural development, how m^6^A mRNA methylation contributes to neurological disorders is largely unexplored. Alzheimer’s disease (AD) is one of the most prevalent human neurodegenerative diseases in the elderly, and synaptic changes are widely regarded as disease-causative. The salient clinical feature of AD is progressive decline of memory function, leading to impaired cognitive function [26]. However, the pathogenesis of AD remains unclear. Recent studies have shown that m^6^A methylation deficiency impairs hippocampal excitatory synaptic transmission [58, 61]. Studies of an AD mouse model (APP/PS1 transgenic mice) have revealed that the mice display increased m^6^A methylation in the cortex and hippocampus, and that expression of *Mettl3* is increased whereas *Fto* expression is reduced in the AD mice [62, 63]. These studies support that dysregulation of the *Mettl3*/*Fto* axis may alter global patterns of m^6^A methylation of mRNAs, further impacting dendritic development, synaptic growth, synaptic assembly, axon guidance, and long-term potentiation, thereby potentially linking m^6^A epitranscriptomic regulation and neurological disorders.

Apart from AD, some genetic variants of *FTO* have been linked to major depressive disorder (MDD) [64, 65]. Moreover, *YTHDC2* has been reported as a potential risk factor for autism spectrum disorder (ASD) in east Asian populations [65, 66]. Integrative analysis of genome-wide association studies (GWAS) for m^6^A single nucleotide polymorphisms (SNPs) has also highlighted potential causal genes important in neurodegenerative disease [67, 68]. For example, *ALKBH5* is associated with various clinical features of MDD, including anxiety, retardation, and cognitive dysfunction [67, 68]. Those studies also suggest that m^6^A may contribute to various other neurodevelopmental and neurodegenerative diseases.

## Concluding remarks and future perspectives

The field of epitranscriptomics has emerged rapidly in recent years, and increasing numbers of researchers are tackling its implications for human disease. In this review, we have summarized m^6^A-mediated epitranscriptomic gene regulation and the mechanisms involved in neural development and neurodegenerative disease. Dynamic m^6^A-dependent transcriptomic regulation has been demonstrated to be involved in embryonic and postnatal neural development, with activities ranging from NSC establishment to maintenance of adult neuronal function. However, we still lack a complete map of the global m^6^A transcriptome during neural development and degeneration in different parts of the CNS. In particular, how m^6^A contributes to neural disorders remains unclear. Fortunately, site-specific m^6^A incorporation in distinct cellular compartments is now feasible since nucleus-localized dCas13 can be fused with a truncated METTL3 methyltransferase domain or cytoplasm-localized Cas can be fused with a modified METTL3/14 methyltransferase complex [69]. We envision these approaches will provide researchers with a powerful arsenal for dissecting cause-and-effect relationships that will reveal the importance of m^6^A in neurological disorders, including AD, Parkinson's disease, and amyotrophic lateral sclerosis (ALS).

How a complete protein-RNA regulome participants are involved in m^6^A mRNA modification remain to be uncovered, and whether its dynamic regulation is cell context-dependent remains to be characterized in detail. Currently, epitranscriptome mapping technologies primarily rely on the sensitivity and specificity of m^6^A modification-specific antibodies. However, it remains challenging to detect a specific RNA modification at single-nucleotide resolution with low background signal. Improvements in detection techniques enabling characterization (quantitatively and at the genome-wide scale) of the distribution of RNA modifications at nucleotide resolution and with greater sensitivity and accuracy will greatly help us understand this critical RNA modification. Antibody-independent approaches have recently been reported [70–72], which will greatly help establish the m^6^A epitranscriptomic landscape in various cellular and developmental contexts with single base resolution. These novel cutting-edge techniques will ultimately facilitate experiments that can characterize m^6^A epitranscriptomic marks in vivo and confirm their regulatory functions at the single cell level.

Finally, the crosstalk between epitranscriptomics and epigenetics is emerging as a new regulatory axis [19]. Some studies have suggested that m^6^A could directly or indirectly regulate chromatin-mediated transcription and accessibility by controlling chromatin regulatory complexes and long noncoding RNAs [5, 19, 73]. Whether or not m^6^A regulates chromatin states is an interesting research avenue that may reveal mechanisms by which epigenetic regulation can contribute to gene regulation during neural development. Such investigations may also help us to better understand if m^6^A modification may control the protein levels of genes involved in neurodegenerative diseases and aging [63]. Our review provides a brief overview of the roles of m^6^A in neural development and degeneration. We believe we are only beginning to understand the mysterious roles of RNA modification in the CNS.

## Data Availability

Not applicable.
